# Polysomnographic correlates of sleep disturbances in de novo, drug naïve Parkinson’s Disease

**DOI:** 10.1007/s10072-021-05622-3

**Published:** 2021-09-29

**Authors:** Beatrice Orso, Francesco Famà, Laura Giorgetti, Pietro Mattioli, Andrea Donniaquio, Nicola Girtler, Andrea Brugnolo, Federico Massa, Enrico Peira, Matteo Pardini, Silvia Morbelli, Flavio Nobili, Dario Arnaldi

**Affiliations:** 1 Department of Neuroscience, Rehabilitation, Ophthalmology, Genetics, Maternal and Child Health (DINOGMI), University of Genoa, Genoa, Italy; 2 IRCCS Ospedale Policlinico San Martino, Genoa, Italy; 3 Department of Health Science (DISSAL), University of Genoa, Genoa, Italy; 4 National Institute of Nuclear Physics (INFN), Genoa section, Via Dodecaneso 33, 16146 Genoa, Italy

**Keywords:** Parkinson’s Disease; Sleep disturbances, Sleep disorders, Polysomnography

## Abstract

**Background:**

Sleep disturbances are common non-motor symptoms of Parkinson’s Disease (PD).

**Methods:**

The aim of this study was to investigate the polysomnographic correlates of sleep changes, as investigated by the Parkinson’s Disease Sleep Scale-2 (PDSS-2), in a cohort of sixty-two consecutive de novo, drug naïve PD patients (71.40 ± 7.84 y/o).

**Results:**

PDSS-2 total score showed a direct correlation with stage shifts (p = 0.008). Fragmented sleep showed an inverse correlation with sleep efficiency (p = 0.012). Insomnia symptoms showed an inverse correlation with wake after sleep onset (p = 0.005) and direct correlation with periodic leg movements (p = 0.006) and stage shift indices (p = 0.003). Motor Symptoms showed a direct correlation with Apnoea-Hypopnoea (AHI; p = 0.02) and awakenings indices (p = 0.003). Dream distressing showed a direct correlation with REM without atonia (RWA, p = 0.042) and an inverse correlation with AHI (p = 0.012). Sleep quality showed an inverse correlation with RWA (p = 0.008).

**Conclusion:**

PDSS-2 features are significantly correlated with polysomnography objective findings, thus further supporting its reliability to investigate sleep disturbances in PD patients.

## Introduction

Sleep disturbances are among the most common non-motor symptoms of Parkinson’s Disease (PD) [1]. These disturbances mostly include insomnia (i.e., difficulties in both falling and staying asleep), fragmentation of sleep, excessive daytime sleepiness and abnormal movements and behaviours, such as periodic limb movements (PLMs) and rapid eye movement sleep behaviour disorder (RBD) [2–4].

These symptoms may manifest early on in the disease course; for instance, sleep fragmentation has been associated with a higher risk of Lewy-body pathology in elderly individuals without clinical PD [5]. Moreover, at least 70% of subjects with idiopathic RBD develop an alpha-synucleinopathy over time [6, 7]. However, besides sleep fragmentation and RBD, several other sleep disturbances are often present in PD patients, at any stage of the disease, affecting patients’ quality of life. Thus, validated and cost-effective tools to reliably investigate sleep disturbances in PD patients are crucial.

Polysomnography (PSG) is regarded as the gold standard to assess sleep dysfunction in PD [8]. However, PSG, especially types I/II PSG (i.e., with electrodes for the full sleep staging), is not widely available, is expensive, and requires technicians and physicians with specific expertise in sleep medicine. Several questionnaires have been published for the clinical assessment of sleep disturbances, and the Parkinson’s Disease Sleep Scale-2 (PDSS-2) is one of the most used tools in PD patients [9]. However, whether and how the PDSS-2 reported features are associated with the PSG objective findings is still unknown.

Thus, the aim of this study was to investigate the PSG correlates of sleep changes, as investigated by the PDSS-2, in a cohort of consecutive de novo, drug naïve PD patients.

## Methods

### Subjects

Sixty-two consecutive drug-naïve outpatients with de novo PD diagnosed according to current criteria [10] were prospectively evaluated. All patients had nigro-striatal dopaminergic impairment demonstrated by ^123^I-FP-CIT-SPECT and diagnosis confirmation at least by a one-year follow-up. Baseline clinical evaluation included the Movement Disorder Society-sponsored revision of the unified Parkinson’s Disease rating scale (MDS-UPDRS), the Mini-Mental State Examination (MMSE), the 15-item geriatric depression scale (GDS-15) and a comprehensive neuropsychological assessment including at least two tests within each of the main cognitive domains (i.e., attention and working memory, executive, language, memory, and visuospatial) [11]. The presence of mild cognitive impairment (MCI) was evaluated according to current criteria, by level-2 assessment [12]. The main exclusion criteria were the presence of neuropsychiatric comorbidities and/or dementia as well as any other medical condition or drug treatment potentially able to interfere with sleep quality. Mild depressive symptoms were not an exclusion criterion. Magnetic resonance imaging or computed tomography were used to rule out brain lesions; the presence of white matter hyperintensities was not an exclusion criterion if the Whalund score was < 2 at each site.

All participants signed an informed consent form in compliance with the Helsinki Declaration of 1975 at the time of evaluation.

### Polysomnographic recording

Within 3 months since diagnosis, patients underwent overnight polysomnography (Somté PSG – Compumedics), performed by technicians with expertise in the field; the sleep scoring was performed following current criteria [13]. PSG derivations were placed according to recommended rules [13] in order to evaluate sleep features, respiratory, cardiac, and limb events. If used, patients were asked to withdraw melatonin, hypnotic medications and antidepressant drugs for two weeks before the recording.

Chin electromyography (EMG) was used to visually quantify REM sleep without atonia (RWA) and the ‘*any*’ REM percentage was used [14]. The following PSG indices were also used for statistical analyses: sleep latency (SL, minutes); wake after sleep onset (WASO, minutes); sleep efficiency (SE, percentage); number of awakenings, normalized to total sleep time (TST); number of stage shifts, normalized to TST; apnoea/hypopnea index (AHI); periodic leg movements index (PLMI).

### Sleep disturbances assessment

Sleep disturbances were assessed using the Italian version of the PDSS-2 scale [15], a 15-item scale with a total score ranging from 0 (no disturbance) to 60 (maximum nocturnal disturbance). Items were then clustered into five groups, namely: motor symptoms, sleep quality, dream distressing, fragmented sleep, and insomnia symptoms [15].

### Statistical analysis

Normal distribution of variables was checked using Shapiro–Wilk test. A Generalized Linear Model (GLM) was applied to investigate the correlations between PDSS-2 scores and PSG indices, adjusting for age, MMSE and MDS-UPDRS-III scores. Statistical threshold was set at p < 0.05. Statistical analyses were performed using Stata13 (StataCorp. 2013. Stata Statistical Software: Release 13. College Station, TX: StataCorp LP). The p-values were corrected using the Benjamini–Hochberg false discovery rate (FDR) approach.

## Results

**Fig. 1:**
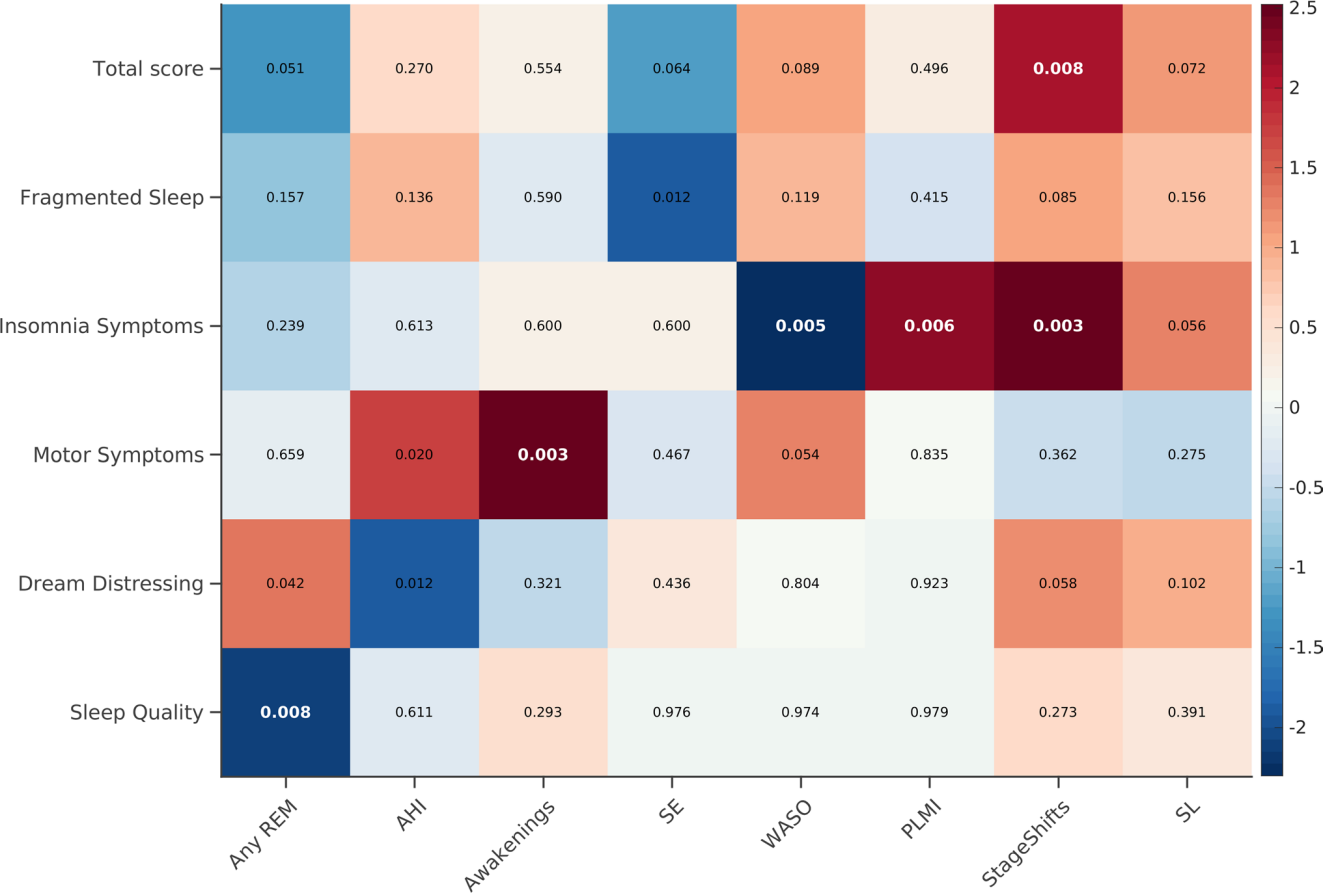
GLM analyses results. GLM direct (red to white) or inverse (blue to white) correlations are shown. The p-values for each correlation are reported. The colour bar represents the correlation coefficients. The p-values that survived the FDR correction are reported in bold white. All analyses were adjusted for age, MMSE and MDS-UPDRS-III. AHI = Apnoea-hypopnea index; GLM = Generalized linear model; MDS-UPDRS-III = Movement Disorders Society-sponsored revision of the Unified Parkinson’s Disease Rating Scale, motor section; MMSE = Mini Mental State Examination; PDSS-2 = Parkinson’s Disease Sleep Scale-2; PLMI = Periodic leg movements index; REM = Rapid eye movement; SE = Sleep efficiency; SL = Sleep latency; WASO = Wake after sleep onset

Main demographic, clinical and PSG data of the PD patients are summarized in Table 1. Main results of the GLM analyses are summarized in Fig. 1.

**Table 1:** Demographic and clinical characteristics of PD patients. Values are shown as mean ± standard deviation

	PD patients
	n = 62
Age (yr)	71.40 ± 7.84
Education (yr)	11.26 ± 3.78
Gender (M:F)	36:26
MMSE score	28.46 ± 1.98
MCI (Y:N)	29:33
MDS-UPDRS-III score	20.76 ± 8.31
GDS-15 score	3.61 ± 3.10
PDSS-2 scores	
Total score	13.91 ± 9.47
Abnormal total score (≥ 18)	n = 16 (25.8%)
Fragmented sleep	4.5 ± 2.45
Insomnia symptoms	1.5 ± 1.79
Motor symptoms	4.24 ± 4.62
Dream distressing	1.06 ± 1.78
Sleep quality	2.61 ± 2.11
Polysomnographic data	
SE (%)	79.42 ± 10.4
*Any-*REM (%)	22.22 ± 44.09
PSG-confirmed RBD	n = 34 (54.83%)
WASO (min)	96.88 ± 72.27
SL (min)	40.28 ± 88.77
Awakenings (n/TST)	0.06 ± 0.04
Stage Shift (n/TST)	0.46 ± 0.19
AHI	12.33 ± 26.33
Mild: 5 < AHI > 15	n = 10 (16.12%)
Moderate: 15 < AHI > 30	n = 8 (12.9%)
Severe: AHI > 30	n = 11 (17.74%)
PLMI	5.38 ± 11.8
PLMI > 5	n = 5 (8.06%)
PLMI > 15	n = 24 (38.7%)

**Legend:** *AHI*, Apnoea-Hypopnea Index; *F*, female; *GDS*, 15-item Geriatric Depression Scale; *M*, male; *MDS-UPDRS-III*, Movement Disorders Society-sponsored revision of the Unified Parkinson’s Disease Rating Scale, motor section; *MMSE*, Mini Mental State Examination; *PD*, Parkinson’s Disease; *PDSS-2*, Parkinson’s Disease Sleep Scale; *PLMI*, Periodic Leg Movement Index; *RBD*, REM sleep behaviour disorder; *REM*, Rapid eyes movements; *SE*, Sleep Efficiency; *SL*, Sleep Latency; *TST*, Total sleep time; *WASO*, Wake After Sleep Onset

Briefly, the PDSS-2 total score showed a significant direct correlation with the Stage Shift index. Fragmented sleep showed a significant inverse correlation with the SE indices that although did not survive to the FDR correction. Insomnia symptoms showed a significant inverse correlation with WASO, a significant direct correlation with Stage Shift and PLMI indices and tended to directly correlate with SL. Motor symptoms showed a significant direct correlation with awakenings and AHI indices and tended to directly correlate with WASO, the AHI correlation did not survive to the FDR correction. Dream distressing showed a significant direct correlation with *Any*-REM index, a significant inverse correlation with AHI and tended to directly correlate with stage shifts, correlations did not survive to the FDR correction. Sleep quality showed a significant inverse correlation with *Any*-REM index.

Moreover, we expand our results exploring the relationship between sleep disturbances (25.8% of patients presented with an abnormal PDSS-2 score) and non-motor symptoms (i.e., hyposmia, constipation, orthostatic hypotension). We did not find any significant difference between PD patients with and without sleep disturbances (Table 2). Furthermore, we did not find any significant difference in the PDSS-2 indices between tremor-dominant (n = 48) and akinetic (n = 14) PD patients (Table 3).

**Table 2:** Non-motor symptoms in patients with and without sleep disturbances, according to the PDSS-2 total score

	Abnormal PDSS-2 (n = 16)	Normal PDSS-2 (n = 46)	p-value
Hyposmia	12 (75%)	36 (78.26%)	0.716
Constipation	13 (81.25%)	26 (56.52%)	0.080
Orthostatic hypotension	4 (25%)	8 (17.39%)	0.72

**Table 3:** Demographic and clinical characteristic of Akinetic and tremor-dominant PD patients. Values are shown as mean ± standard deviation

	Akinetic (n = 48)	Tremor-dominant (n = 14)	p-value
Age	71.20 ± 5.47	72.07 ± 6.54	0.71
Education	11.28 ± 3.67	11.29 ± 4.19	1
MMSE	28.51 ± 2.15	28.21 ± 1.93	0.62
PDSS-2 score			
Total score	13.26 ± 9.02	15.78 ± 10.84	0.38
Motor symptoms	3.91 ± 4.22	5.07 ± 5.90	0.41
Sleep quality	2.51 ± 1.90	2.92 ± 2.73	0.51
Dream distressing	0.87 ± 1.34	1.85 ± 2.76	0.069
Fragmented sleep	4.42 ± 2.55	4.57 ± 2.13	0.84
Insomnia symptoms	1.53 ± 1.82	1.35 ± 1.64	0.75

## Discussion

We investigated the clinical and polysomnographic correlates of sleep disorders in a naturalistic group of consecutive de novo, drug naïve PD patients, thus providing a reliable characterization of such disturbances. Indeed, most studies investigating sleep disturbances in PD were conducted in selected group of patients (for instance, only those with sleep complaints) and without the support of full polysomnography.

Overall, 25.8% of our patients presented with sleep disturbances, according to the PDSS-2 total score. Moreover, 54.8% of patients had PSG-confirmed RBD, 46.8% had clinically significant AHI (i.e., more than 5), and 38.7% had clinically significant PLMI (i.e., more than 15), according to international criteria applied to the PSG data [16].

The PDSS-2 total score was directly correlated with the Stage Shift PSG index, as to say that the amount of shifting in sleep phases is directly affected by the presence of sleep disturbances. Indeed, fragmented sleep and insomnia symptoms represent a major complaint among PD patients [2, 5, 17]. Accordingly, in our group insomnia, as investigated with the PDSS-2, was significantly correlated with reduced WASO, sleep efficiency and PLMI indices, as well as with prolonged sleep latency, that is, our patients mostly suffered from sleep-onset insomnia, rather than sleep-maintenance insomnia. Moreover, insomnia symptoms were also significantly correlated with stage shifts and PLMI, which are known to be associated with each other. Indeed, in PD patients, it has been shown that motor symptoms, i.e. PLM, restless legs or arms at night and limbs’ muscle cramps, might cause sleep fragmentation [18], which can be perceived as insomnia by patients.

We also found that motor symptoms correlated with the awakenings index and AHI, although not surviving the FDR correction. Despite this, sleep-related breathing disorders have been associated with increased arousability [19] and subsequent awakenings [20], and both factors might cause the perception of moving during night time in affected patients.

As expected, dreaming distress, which is the clinical complaint of RBD, showed a direct correlation trend with chin-RWA (i.e. any-REM), which is the PSG finding of RBD, as well as an inverse correlation with AHI. Interestingly, chin-RWA was also indirectly correlated with sleep quality, suggesting that the presence and the severity of RBD is associated with the perception of reduced sleep quality in PD patients. Finally, considering that sleep-related breathing disorders increase sleep fragmentation [20], they may also reduce REM sleep duration, thus reducing the opportunity of perceiving the dreaming distress.

The main strength of the present study is represented by the large naturalistic cohort of de novo, drug-naïve PD patients undergoing full polysomnography. A limitation of the study is that a video recording was not performed, thus we could not investigate abnormal sleep behaviours. Moreover, we decided to include only patients in an early stage of the disease and without dopaminergic treatment to limit confounders. Thus, the present results on polysomnographic features should be regarded as indicative in early PD, but not necessarily for more advanced stages and under dopaminergic medication.

In conclusion, this study shows that the specific features of the PDSS-2 are significantly associated with PSG objective findings and thus supports the PDSS-2 reliability to investigate sleep disturbances in PD patients.

## Data Availability

The datasets generated during and/or analysed during the current study are available from the corresponding author on reasonable request.
